# Cantharidin Induces Apoptosis and Promotes Differentiation of AML Cells Through Nuclear Receptor Nur77-Mediated Signaling Pathway

**DOI:** 10.3389/fphar.2020.01321

**Published:** 2020-08-28

**Authors:** Zanyang Yu, Li Li, Chengqiang Wang, Hui He, Gen Liu, Haoyue Ma, Lei Pang, Mingdong Jiang, Qianwei Lu, Pan Li, Hongyi Qi

**Affiliations:** ^1^ College of Pharmaceutical Sciences, Southwest University, Chongqing, China; ^2^ Radiotherapy Department, Chongqing Ninth People’s Hospital, Chongqing, China

**Keywords:** cantharidin, Nur77, acute myeloid leukemia, differentiation, apoptosis

## Abstract

**Background:**

Acute myeloid leukemia (AML) is a hematopoietic malignancy characterized by uncontrolled proliferation and accumulation of myeloblasts in the bone marrow (BM), blood, and other organs. The nuclear receptors Nur77 is a common feature in leukemic blasts and has emerged as a key therapeutic target for AML. Cantharidin (CTD), a main medicinal component of Mylabris (blister beetle), exerts an anticancer effect in multiple types of cancer cells.

**Purpose:**

This study aims to characterize the anti-AML activity of CTD *in vitro* and *in vivo* and explore the potential role of Nur77 signaling pathway.

**Study Design/Methods:**

The inhibition of CTD on cell viability was performed in different AML cells, and then the inhibition of CTD on proliferation and colony formation was detected in HL-60 cells. Induction of apoptosis and promotion of differentiation by CTD were further determined. Then, the potential role of Nur77 signaling pathway was assessed. Finally, anti-AML activity was evaluated in NOD/SCID mice.

**Results:**

In our study, CTD exhibited potent inhibition on cell viability and colony formation ability of AML cells. Moreover, CTD significantly induced the apoptosis, which was partially reversed by Z-VAD-FMK. Meanwhile, CTD promoted the cleavage of caspases 8, 3 and PARP in HL-60 cells. Furthermore, CTD obviously suppressed the proliferation and induced the cell cycle arrest of HL-60 cells at G2/M phase. Meanwhile, CTD effectively promoted the differentiation of HL-60 cells. Notably, CTD transiently induced the expression of Nur77 protein. Interestingly, CTD promoted Nur77 translocation from the nucleus to the mitochondria and enhanced the interaction between Nur77 and Bcl-2, resulting in the exposure of the BH3 domain of Bcl-2, which is critical for the conversion of Bcl-2 from an antiapoptotic to a proapoptotic protein. Importantly, silencing of Nur77 attenuated CTD-induced apoptosis, reversed CTD-mediated cell cycle arrest and differentiation of HL-60 cells. Additionally, CTD also exhibited an antileukemic effect in NOD/SCID mice with the injection of HL-60 cells into the tail vein.

**Conclusions:**

Our studies suggest that Nur77-mediated signaling pathway may play a critical role in the induction of apoptosis and promotion of differentiation by CTD on AML cells.

## Introduction

Leukemia is a kind of malignant clonal disease originating from hematopoietic stem cells. It is characterized by uncontrolled hematopoietic cell proliferation, differentiation disorder, and inhibition of apoptosis leading to normal blood cell reduction (Banta et al., 2018). Acute myeloid leukemia (AML) is the most common acute leukemia in adults. AML incidence is age-dependent, rising markedly in patients aged ≥60 years. In Europe, ageing of the population contributes to the increase in AML incidence from 3.48 in 1976 to 5.06 patients per 100,000 people in 2013 (Roman et al., 2016). In China, there were 75,300 newly increased cases and 53,400 leukemia patient deaths in 2015 (Chen et al., 2016). In 2019, the United States is expected to add 61,780 leukemia patients and 22,840 deaths. Among them, AML patients are expected to add 21,450 new cases and 10,920 patient deaths. In terms of morbidity and mortality statistics, the number of men is higher than women (Siegel et al., 2019). At present, AML efficacy is greatly limited by severe side effects, multiple drug resistance, and expensive costs (Burnett et al., 2011; O’Donnell et al., 2017).

The orphan nuclear receptor subfamily (NR4A) is a family of three highly homologous orphan nuclear receptors with a variety of physiological and pathological effects that are reported to be dysregulated in a variety of cancer types (Beard et al., 2015; Banta et al., 2018). The orphan nuclear receptor Nur77 is a protein encoded by NR4A1 and plays an important regulatory role in cell proliferation, apoptosis, differentiation, inflammation, atherosclerosis, metabolism, DNA repair, and tumorigenesis (Chintharlapalli et al., 2005; Lee et al., 2009; Boudreaux et al., 2012). Accumulating evidence has shown that Nur77 plays a dual role in promoting or inhibiting tumors depending on the specific cellular environment. It is reported that Nur77 is overexpressed in a variety of cancer cells and tissue samples, resulting in increased proliferation and survival in these cells and tissues, such as: prostate cancer (Wu et al., 2018), breast cancer (Xie et al., 2016), and so on. In recent years, Nur77 has been found to be an important tumor suppressor gene in AML. The expression loss of Nur77 is a common feature of AML patients (Boudreaux et al., 2012). Targeted activation of Nur77 expression has been shown as a potential novel approach for intervention in the treatment of AML (Boudreaux et al., 2019).

Mylabris, the dried body of blister beetle (*Mylabris phalerata* and *M. cichorii*), has been used in traditional Chinese medicine (TCM) for more than 2,000 years. Cantharidin (CTD) is found to be the major bioactive component of mylabris. In recent studies, it has been found that CTD and its derivatives, such as Cantharidic acid and norcantharidin, inhibited leukemia cell proliferation, induced apoptosis, caused cell cycle arrest, and potentiated the inhibitory effects of chemotherapy drugs (, 2001; Dorn et al., 2009; Liao et al., 2011; Sun et al., 2016; Wang et al., 2018). However, the potential molecular mechanisms are still not fully understood. Thus, it is interesting for us in this study to discover whether Nur77-related signaling pathway plays an important role in the anti-AML activity of CTD, which may provide the evidence for the traditional use of mylabris in cancer treatment.

In the current study, we characterized the effect of CTD on apoptosis and differentiation of HL-60 cells, and the potential role of Nur77-mediated signaling pathway was carefully investigated.

## Materials and Methods

### Chemicals and Reagents

CTD (purity ≥98%, product number: MB6525) was purchased from Dalian Meilun Biotechnology Co., Ltd. and added with an appropriate amount of DMSO solution to dissolve into a 32 mM stock solution, stored at −20°C for further use. The antibodies against caspase 3, cleaved caspase 3, cleaved caspase 8, Cyclin E, Cyclin B1, CDK2, p21, p27, and p53 were obtained from Wanlei Biotechnology (Shenyang, China). The antibodies against Nur77, PARP, cleaved PARP, c-Jun, Jun-B, Bcl-2, and Bax were purchased from Santa Cruz Biotechnology (CA, USA). The antibodies against *β*-actin, mouse, or rabbit IgG were obtained from Sigma-Aldrich (St. Louis, MO, USA). Hoechst 33342 was obtained from Wanlei Biotechnology (Shenyang, China). Other chemicals were obtained from Sigma-Aldrich, unless indicated otherwise.

### Cell Culture

AML cell lines HL-60, Kasumi-1, and OCL-AML3 and human normal 293T and HUVEC cells were purchased from the Cell Bank of Chinese Academy of Sciences (Shanghai, China). AML cells were grown in RPMI-1640 medium (Gibco, Life Technologies, Carlsbad, CA, USA) supplemented with 10% fetal bovine serum (FBS) (Invitrogen, USA) and 1% penicillin/streptomycin (Invitrogen, USA). HUVEC cells were cultured in DMEM supplemented with 10% FBS, 2 mM L-glutamine, 1% penicillin/streptomycin. All cells were maintained at 37°C in a 5% CO_2_ and 95% humidified atmosphere.

### Cell Viability Assay

Cell viability was evaluated by the cell counting kit-8 (CCK-8) (Dojindo, Shanghai, China) assay. Cells (8 × 10^4^ cells/ml) were treated with various concentrations of the CTD. After 72 h of incubation, cells were added with 10 μl of CCK-8 for each well. The optical density (OD) was determined at a wavelength of 450 nm using a microplate reader (Biotek, USA). Cell viability was expressed as a percentage of that of the control (untreated) cells.

### Cell Proliferation Assay

Cell Proliferation Assay was determined using trypan blue solution (Sigma, St. Louis, MO, USA). Cells (5× 10^4^ cells/ml) were treated with various concentrations of the CTD separately for 24, 48, 72, 96, 120 h and then stained with trypan blue (0.4%). The viable cells were counted using a hemocytometer.

### Colony Formation Assay

Colony forming units were assayed in methylcellulose (StemCell Technologies, Canada) supplemented with 10% FBS and 1% penicillin/streptomycin. Vehicle or CTD was added to methylcellulose containing 2,500 cells in a 24-well plate. Colonies were evaluated microscopically 14 days after plating.

### Apoptosis and Cell Cycle Analysis by Flow Cytometry

Cells were harvested, washed, and incubated in the binding buffer containing Annexin V-FITC and propidiumiodide (PI) for apoptosis analysis. Cells for cell cycle analysis were fixed, incubated with RNase A, and then stained with PI in the dark. Flow cytometry analyses were performed on a BD LSR Fortessa Cell Analyzer (BD Biosciences, San Jose, CA, USA). Data were analyzed using Flow Jo 7.6.1 software (Tree Star, Inc., Ashland, OR, USA).

### Hoechst 33342 Staining

After treatment, HL-60 cells were centrifuged and collected in Eppendorf tubes. Then, cells were fixed with 4% paraformaldehyde in PBS for 10 min and washed with 1× PBS for three times. Hoechst 33342 (10 mg/ml) stock solution was diluted with 1× PBS (1:2,000) and then added into each well. The plates were protected from light exposure and kept at room temperature for 10 min. Finally, the plates were washed with 1× PBS for three times again. Cells with fluorescence were observed and photographed under a fluorescence microscope (Leica DM4000B, Wetzlar, Germany).

### Wright–Giemsa Staining

Morphological assessment of differentiation was performed using Wright–Giemsa (Leagene, China) staining according to the manufacturer’s protocol on slides prepared by cytospin of HL-60 cells after 96 h treatment. The morphology of cells was examined under a light microscope.

### Nitro Blue Tetrazolium (NBT) Reduction Assay

After 96 h treatment, cells were incubated with NBT solution at 37°C for 30 min. The cells capable of reducing NBT (J&K, Beijing, China) were measured by counting the number of cells containing the precipitated formazan particles in 200 cells of three random visions. 12-O-Tetradecanoylphorbol-13-acetate was used to stimulate the formation of formazan.

### CD11b Detected by Flow Cytometry

Cells were collected and washed twice with ice-cold PBS. Then, cells were incubated with the direct fluorescein isothiocyanate-labeled anti-CD11b antibody (BD Biosciences, San Jose, CA, USA) on ice for 30 min, washed twice with PBS, and the level of antibody binding to the cells was quantified using flow cytometry (BD Biosciences, San Jose, CA, USA).

### Construction and Production of Nur77 shRNA Lentivirus Vector

Nur77 specific small hairpin RNA (shNur77) and shRNA control with nontargeting sequence were constructed based on the lentivirus-based RNAi vector pLKO.1 shRNA/GFP/Neo (GeneChem Co. Ltd.). The human shNur77 target is 5′-TACACAGGAGAGTTTGACA-3′. Lentiviral vectors were packaged through cotransfecting 293T cells with the ViraPower Lentiviral Expression Systems (Invitrogen, USA) following the instructions provided by the manufacturer. After 48 h, the transfection efficiency was monitored. Lentiviral particles were collected within 48–72 h after transfection. Stable cells were then generated as follows: HL-60 cells in 6-well plate were infected with the filtered media containing lentiviruses of shNur77 or nonspecific pLKO.1 shRNA. After 48 h, the media was changed to fresh media containing puromycin and hygromycin B for stable cell selection. Cells were subsequently passed and reached 100% confluence in a 10 cm dish in 3–4 weeks (Wang et al., 2017).

### Animal Studies

This animal experiment was conducted under the supervision of the Southwestern University Institutional Animal Care and Use Committee, in accordance with the approved protocol with the animal ethic clearance proof (ID: XXYL (2018) 05). NOD/SCID mice were purchased from Beijing Vital River Laboratory Animal Technologies Co. Ltd (license number: SCXK (Beijing) 2016-0006). Mice were housed under SPF condition in Southwest University. All experiments conformed to the Guide for the Care and Use of Laboratory Animals published by the US National Institutes of Health (NIH Publication, eighth edition, 2011) and were conducted under the supervision of the Southwestern University Institutional Animal Care and Use Committee, in accordance with the approved protocol. Mice were sublethally irradiated with 2.4 Gy. 24 h later, HL-60 cells were injected into the tail vein (5 × 10^6^ cells/mouse). Mice were randomly divided into three groups (n = 6 per group) respectively. After 7 days, mice in two treatment groups were intravenously given every three days with CTD dissolved in 200 μl of saline at doses of 0.2 mg/kg and 0.4 mg/kg, respectively. Mice in the control group were intravenously given with saline (200 μl). The survival time of each group of mice was recorded. Mice were euthanized 42 days after the injection of HL-60 cells.

### Western Blotting Analysis

Cells were harvested and suspended into 80 ml of lysis buffer containing protease inhibitors (Beyotime, China) and the concentration of the protein extract was determined using the BCA protein assay kit (Beyotime, China). Sixty micrograms of protein was diluted in sample loading buffer and heated at 95°C for 5 min. The denatured proteins were separated by 12% sodium dodecyl sulfate–polyacrylamide gel electrophoresis and transferred to polyvinylidene difluoride membranes. The membranes were blocked for 40 min at room temperature in tris-buffered saline containing 5% BSA and 0.1% tween 20, followed by overnight incubation at 4°C, separately, with specific primary antibodies which detect proteins involved in various cell signaling pathways. The membranes were washed and incubated with the appropriate secondary antibody at room temperature for 1 h. Blots were visualized using a standard enhanced chemiluminescence system (Bio-Rad Labs, USA).

### Immunohistochemistry, and Hematoxylin–Eosin (HE) Staining

Immunohistochemistry and HE staining were performed as described previously (Giunta et al., 2015; Wang et al., 2017).

### Statistical Analysis

All data were presented as the mean ± SD. The significant difference in the study was examined using Student’s t test or one-way ANOVA. A p-value of less than 0.05 was considered to be significant. All calculations were performed using Prism 5.03 (GraphPad Software Inc., San Diego, CA, USA).

## Results

### CTD Inhibited Cell Growth of AML Cells

**Figure 1:**
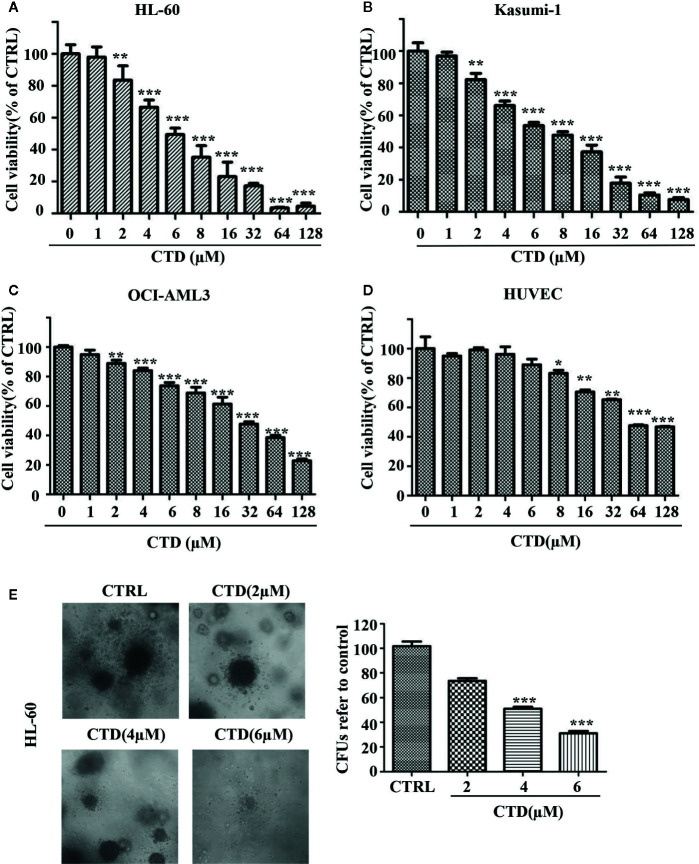
CTD inhibited the growth of AML cells. **(A–D)** HL-60, Kasumi-1, OCI-AML3, and HUVEC cells were treated with CTD as indicated for 72 h. Cell viability was measured using CCK-8 assay. **(E)** HL-60 cells were cloned in methylcellulose and treated with CTD as indicated. Two weeks later, colonies >50 μm in diameter were counted. The colony images were a representative of three independent experiments. Values are presented as the means ± SD. *p < 0.05, **p < 0.01, and ***p < 0.001 vs control.

To investigate whether CTD has an inhibitory effect on AML cells, we first measured the viability of three AML cell lines after CTD treatment for 72 h with CCK-8 assay. As shown in **Figures 1A–C**, CTD significantly inhibited the viability of HL-60, Kasumi-1 and OCI-AML3 cells. Notably, HL-60 (IC_50_ = 6.21 μM) and Kasumi-1 (IC_50_ = 8.00 μM) cells are more sensitive to CTD than OCI-AML3 (IC_50 =_ 2 8.70 μM) cells. In view of the high activity against AML cells, CTD was further investigated to evaluate its selectivity against AML cells over normal cells. The results in **Figure 1D** indicated that CTD exhibited relatively weak inhibition on the viability of HUVEC cells with IC50 value of 75.63 μM. The selectivity index for HL-60 and normal cells was 12.2, which indicated that CTD could selectively eliminate AML cells with relatively low toxicity against normal cells. To investigate the effect of CTD on the colony formation ability of AML cells, HL-60 cells were treated with different concentrations of CTD (0, 2, 4, 6 μM) for 14 days. As shown in **Figure 1E**, the number of colonies formed by HL-60 cells was remarkably suppressed by 4 and 6 μM of CTD compared with control (*p* < 0.001). Morphologically, the size of colonies also obviously reduced after 4 and 6 μM of CTD treatment.

### CTD Induced Apoptosis in HL-60 Cells

**Figure 2:**
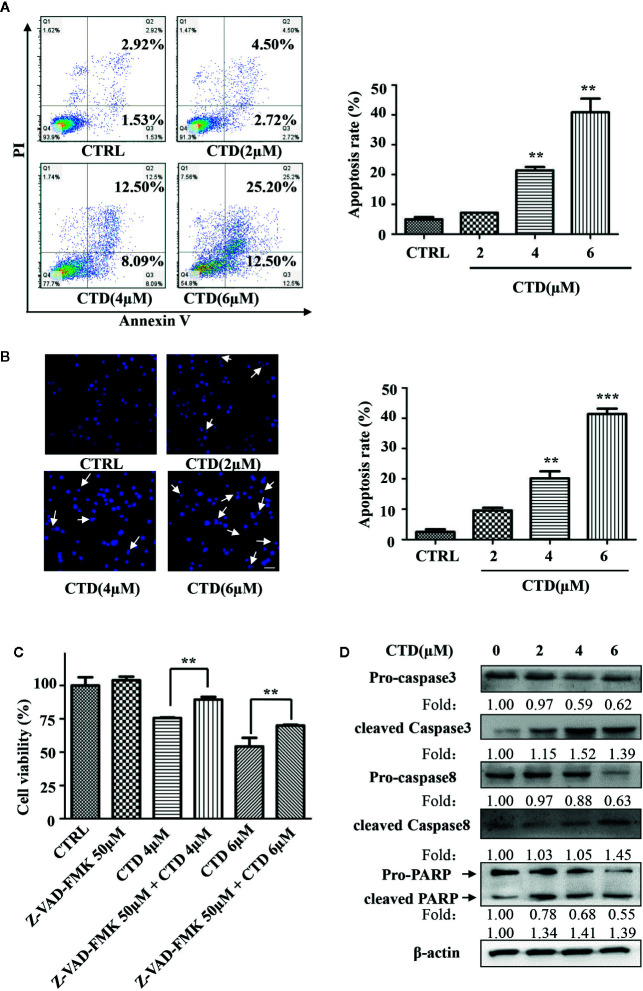
CTD induced apoptosis of HL-60 cells. HL-60 cells were treated with CTD as indicated for 48 h. **(A, B)** Apoptotic cells were determined by flow cytometry and Hoechst 33342 staining (n = 3). **(C)** HL-60 cells were pre-treated with the pan-caspase inhibitor Z-VAD-FMK for 2 h and then treated with CTD as indicated for 48 h. Cell viability was measured using CCK-8 assay. **(D)** HL-60 cells were treated with CTD as indicated for 48 h and then apoptosis-related proteins were detected by Western blotting. The blots were a representative of three independent experiments. The scale bar is 100 μm. Values are presented as the means ± SD. **p < 0.01, ***p < 0.01 vs control.

To determine whether the inhibition of viability of HL-60 cells by CTD was attributed to the activation of apoptotic cell death, we evaluated the apoptosis level by flow cytometry with Annexin V/PI staining. As shown in **Figure 2A**, 4 and 6 μM of CTD significantly induced the apoptosis of HL-60 cells. Then, we further determined the apoptosis by Hoechst 33342 staining. After treatment with CTD, many more cells with condensed and fragmented nuclei were observed in HL-60 cells treated with 4 and 6 μM of CTD than those in the control (**Figure 2B**). As caspase-mediated apoptosis is one of the most common apoptosis pathways, we then determined the influence of Z-VAD-FMK, a pan-caspase inhibitor, on the apoptosis induced by CTD. **Figure 2C** showed that Z-VAD-FMK (50 μM) significantly mitigated the inhibitory effect of CTD (4 and 6 μM) on the viability of HL-60 cells (*p* < 0.01). Furthermore, several apoptosis-relevant proteins were determined by western blotting after HL-60 cells treated with CTD for 48 h. **Figure 2D** indicated that CTD obviously reduced the level of pro-caspase 3, pro-caspase 8, and pro-PARP and enhanced the level of cleaved-caspase 3, cleaved-caspase 8, and cleaved-PARP.

### CTD Caused Cell Cycle Arrest of HL-60 Cells

**Figure 3:**
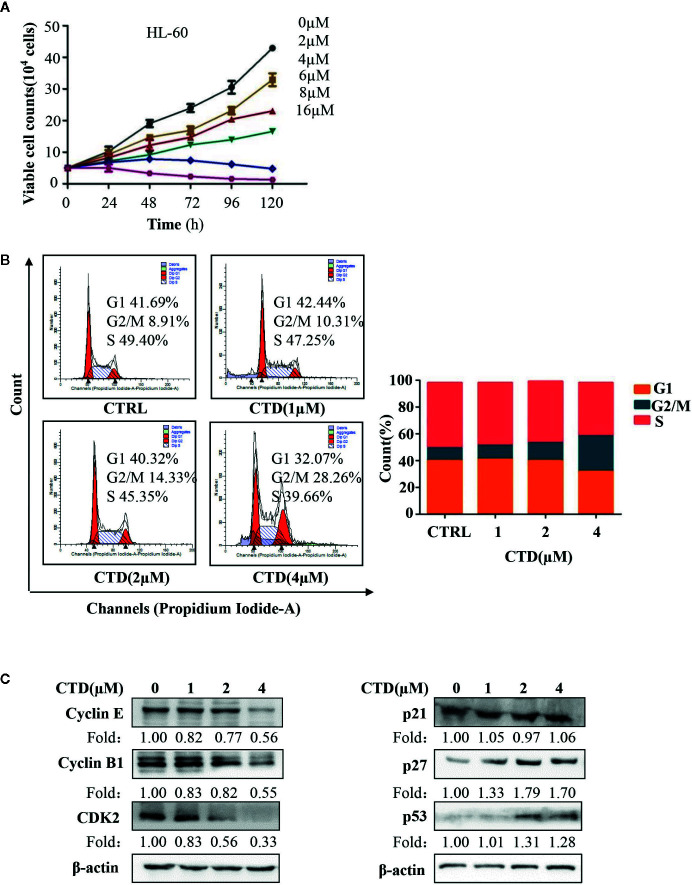
CTD suppressed proliferation and induced cell cycle arrest in HL-60 cells. **(A)** HL-60 cells were treated with CTD as indicated for 120 h, and cell proliferation assay was performed by trypan blue exclusion. **(B)** HL-60 cells were treated with CTD as indicated for 48 h. After RNase A treatment and PI staining, cell cycle was determined by flow cytometry quantitatively (n = 3). **(C)** The cell cycle related proteins were detected by Western blotting after treatment with CTD as indicated for 48 h. The blots were a representative of three independent experiments. Values are presented as the means ± SD.

In order to determine the effect of CTD on the cycle arrest of HL-60 cells, we first evaluated the influence of CTD on the proliferation of HL-60 cells. The Trypan Blue dye exclusion test was performed in HL-60 cells with CTD treatment for 120 h. As shown in **Figure 3A**, CTD significantly inhibited the proliferation of HL-60 cells in a concentration-dependent manner. Notably, 8 and 16 μM of CTD completely suppressed the proliferation of HL-60 cells. Then, we determined the effect of CTD on the cell cycle distribution of HL-60 cells by flow cytometry with PI staining. **Figure 3B** showed that 4 μM of CTD induced an obvious cell cycle arrest at G2/M phase in HL-60 cells. To further explore the potential mechanisms of CTD on G2/M cell cycle arrest, the expression of cell cycle related proteins was detected by Western blotting after HL-60 cells treated by CTD for 48 h. We found that 4 μM of CTD obviously down-regulated the protein level of cyclin E, cyclin B1, and CDK2, and up-regulated the protein level of p27 and p53 (**Figure 3C**).

### CTD Promoted Differentiation of HL-60 Cells

**Figure 4:**
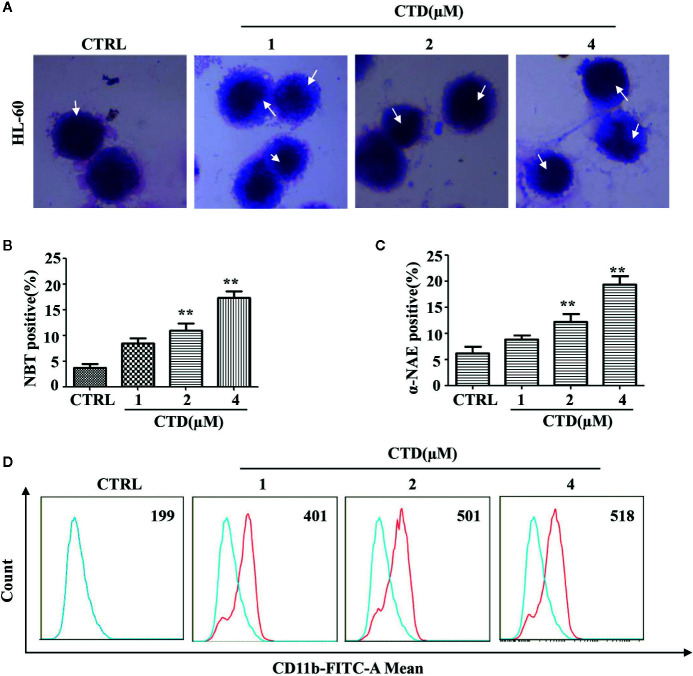
CTD induced differentiation of HL-60 cells. HL-60 cells were treated with CTD as indicated for 96 h and then subjected to **(A)** Wright**–**Giemsa staining, white arrow points to nuclear chromatin condensation. **(B)** NBT reduction staining, **(C)** *α*-NAE staining and **(D)** CD11b determination with flow cytometry (n = 3). **p < 0.01 *vs* control.

To examine the effect of CTD on myeloid differentiation of AML cells, Wright**–**Giemsa staining was first performed to detect the morphologic changes of HL-60 cells treated with CTD for 96 h. As shown in **Figure 4A**, CTD (1 to 4 μM) obviously induced the differentiation of HL-60 cells as evidenced by the change in morphological features such as reduced nuclei/cytoplasm ratio and condensed, distorted, and stab form of nuclei. Moreover, **Figures 4B, C** showed that CTD significantly increased the NBT and *α*-NAE positive cells at 2 and 4 μM (*p* < 0.01). To further characterize the differentiation mediated by CTD, we determined the expression of CD11b, a marker of myeloid differentiation, by flow cytometry. Results in **Figure 4D** showed that CTD obviously increased the fluorescence intensity of CD11b in HL-60 cells with 2.6-fold of increase for 4 μM of CTD.

### Mitochondrial Localization of Nur77 and Subsequent Bcl-2 Transformation Are Involved in CTD-Induced Apoptosis of HL-60 Cells

**Figure 5:**
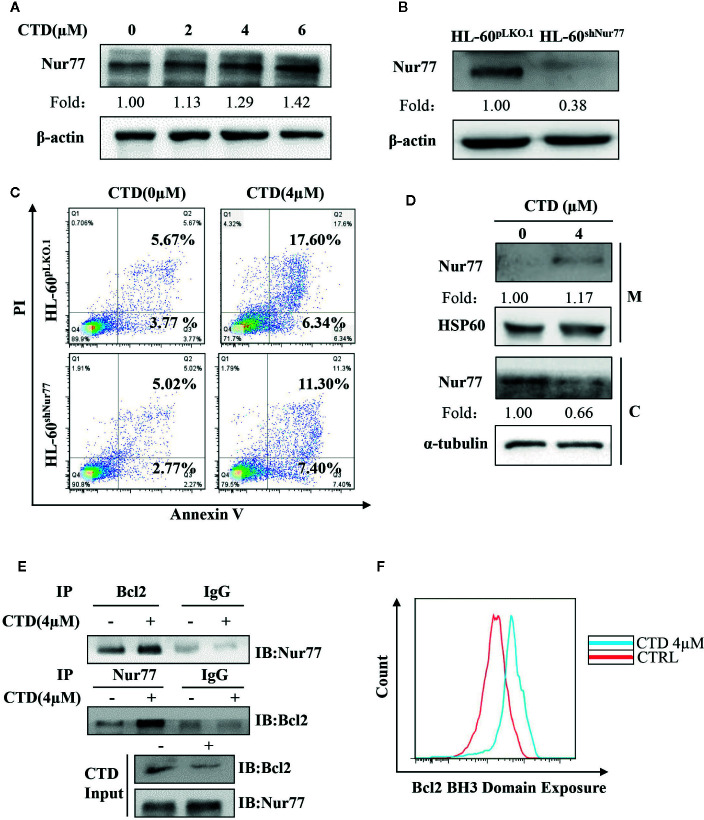
CTD promoted mitochondrial localization of Nur77 and subsequent Bcl-2 transformation. **(A)** HL-60 cells were treated with CTD as indicated for 48 h and then subjected to Western blotting for the detection of Nur77 protein level. **(B)** Western blotting detection of the expression of Nur77 in HL-60^shNur77^ cells and HL-60^ShNC^ cells. **(C)** HL-60^shNur77^ cells and HL-60^pLKO.1^ cells were treated with or without 4 μM CTD for 48 h, respectively. Apoptotic cells were determined by flow cytometry (n = 3). **(D)** After treatment of HL-60 cells with or without 4 μM CTD for 24 h, cell lysates were subjected to Western blotting for analyzing Nur77 and HSP60 in mitochondria (M) and Nur77 and *α*-tubulin in cytoplasm **(C)**. **(E)** HL-60 cells were treated with or without 4 μM CTD for 24 h. Cell lysates were immunoprecipitated with anti-Nur77 or anti-Bcl2 antibody, and then Nur77 and Bcl-2 were detected by Western blotting. Input, cell lysates without IP process is set as a positive control. IgG, IP with anti-immunoglobulin G (IgG) is set as a negative control. The blots are a representative of three independent experiments. **(F)** HL-60 cells were treated with CTD for 24 h and then Bcl2 BH3 Domain Exposure was determined by flow cytometry with an antibody against Bcl-2 (BH3) (n = 3).

Previous studies demonstrate that loss of Nur77 expression is a key pathological factor in the development of AML, and pharmacological target of Nur77 provides a novel approach for the therapeutic intervention in AML (Mullican et al., 2007; Boudreaux et al., 2019). Thus, we determined the influence of CTD on the expression of Nur77. As shown in **Figure 5A**, CTD up-regulated the expression of Nur77 in a concentration-dependent way. To explore the potential role of Nur77 in the anti-AML activity of CTD, we constructed HL-60^shNur77^ cell line and HL-60^pLKO.1^ cell line by stable transfection of HL-60 cells with shNur77 and a pLKO.1 vector, respectively. **Figure 5B** showed that the level of Nur77 in HL-60^shNur77^ cells was significantly lower than that in HL-60^pLKO.1^ cells. Then, we determined the role of Nur77 in CTD-mediated apoptosis. Flow cytometry analysis demonstrated that the apoptosis rate in HL-60^shNur77^ cells with CTD treatment partially reduced than that in HL-60^pLKO.1^ cells with CTD treatment (**Figure 5C**), indicating the critical role of Nur77 in CTD-induced apoptosis. Accumulating evidence indicates that the key mechanism of apoptosis induction by Nur77 is the translocation of Nur77 from the nucleus to the mitochondria where Nur77 binds Bcl-2 to convert it from an antiapoptotic to a proapoptotic protein (Li et al., 2000; Lin et al., 2004; Kolluri et al., 2008; Thompson et al., 2010; Banta et al., 2018). To examine whether CTD can affect the localization of Nur77 to mitochondria, we isolated the mitochondrial protein and cytosolic protein from HL-60 cells with or without CTD treatment. Our results of Western blotting (**Figure 5D**) showed an obvious increase in the level of Nur77 in mitochondria and a decrease in the level of Nur77 in the cytosol, suggesting that CTD promoted the translocation of Nur77 to the mitochondria. To determine the influence of CTD on the interaction between Nur77 and Bcl-2, we performed coimmunoprecipitation after HL-60 cells were treated with CTD or vehicle for 24 h. The results showed that the interaction between Nur77 and Bcl-2 increased in HL-60 cells after CTD treatment (**Figure 5E**). Previous studies indicated that the interaction between Nur77 and Bcl-2 led to the exposure of the BH3 domain in Bcl-2, which is critical for the functional conversion of Bcl-2 (Lin et al., 2004). To determine the effect of CTD on the conformational change of Bcl-2, we performed a flow cytometry with a specific antibody against the BH3 domain of Bcl-2. Our results in **Figure 5F** demonstrate that CTD remarkably enhanced the fluorescence intensity of Bcl-2(BH3) in HL-60 cells treated by CTD for 24 h.

### Nur77 Is Required for CTD-Induced Cell Cycle Arrest and Differentiation of HL-60 Cells

**Figure 6:**
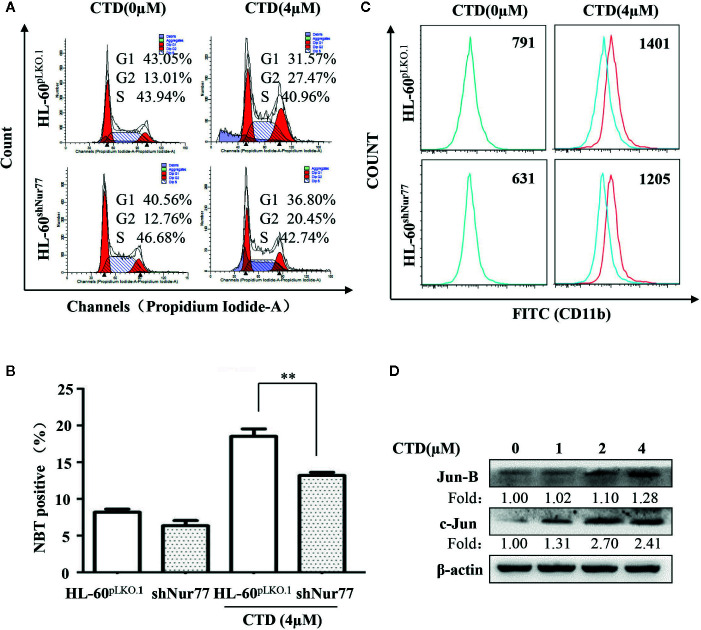
Effect of silencing Nur77 on the cell cycle arrest and differentiation in HL-60 cells. **(A)** HL-60^pLKO.1^ and HL-60^shNur77^ cells were administered with or without 4 μM CTD for 48 h, and then the cell cycle was detected by flow cytometry (n = 3). **(B)** HL-60^pLKO.1^ and HL-60^shNur77^ cells were treated with or without 4 μM CTD for 96 h and then the number of positive cells was counted by the NBT reduction assay. **(C)** HL-60^pLKO.1^ and HL-60^shNur77^ cells were treated with or without 4 μM CTD for 96 h, and then CD11b level was detected by flow cytometry (n = 3). **(D)** HL-60 cells were treated with CTD as indicated for 48 h. Western blot was used to detect the expression of Jun B and c-Jun. The blots are a representative of three independent experiments. **p < 0.01 vs control.

It has been reported that Nur77 plays a key role in the differentiation of AML cells (Mullican et al., 2007). We first determined the potential role of Nur77 in the CTD-induced cell cycle arrest. It can be seen that the percentage of G2/M phase in the CTD-treated HL-60^shNur77^ cells was lower than that in the CTD-treated HL-60^pLKO.1^ cells, with a reduction of 7.02% (**Figure 6A**). Then, we further determined whether Nur77 was involved in the CTD-induced differentiation of HL-60 cells. Our results in **Figure 6B** showed that CTD-treated HL-60^shNur77^ cells exhibited lesser NBT positive cells than CTD-treated HL-60^pLKO.1^ cells (*p* < 0.01). Moreover, the CD11b level in HL-60^shNur77^ cells after CTD treatment for 96 h also obviously reduced compared with that in HL-60^pLKO.1^ cells (**Figure 6C**). Meanwhile, we also determined the level of differentiation-related transcription factors c-Jun and Jun B, which were reported to be the Nur77-mediated downstream mechanism (Mullican et al., 2007). The western blotting results indicate that the expression of Jun B and c-Jun obviously increased in HL-60 cells after treatment with CTD for 48 h (**Figure 6D**).

### Antileukemic Activity Effect of CTD in NOD/SCID Mice

**Figure 7:**
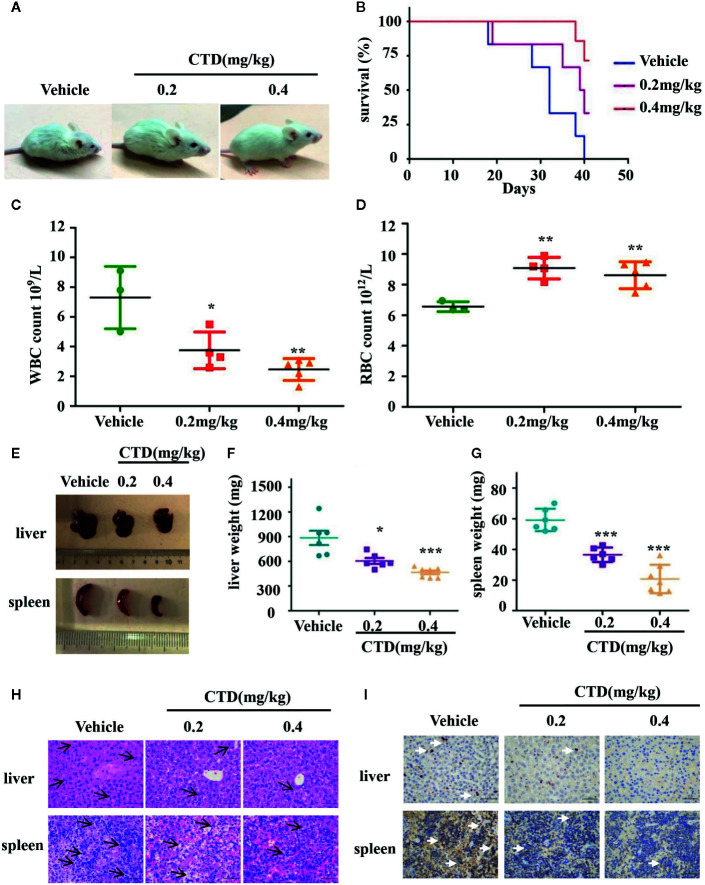
Antileukemic activity of CTD in NOD/SCID mice. Mice were sublethally irradiated with 2.4 Gy and then were randomly divided into three groups as indicated (n = 6). After 24 h, HL-60 cells were injected into the tail vein (5 × 10^7^ cells) of mice in corresponding groups. After one week, CTD (0.2 and 0.4mg/kg) was given *via* intraperitoneal injection, while vehicle group was administrated with normal saline once every 3 days. **(A)** The physical status was compared. **(B)** Kaplan–Meier curves showing overall survival of mice. **(C)** White blood cells (WBC) level; **(D)** Red blood cells (RBC) level. **(E–G)** The weight of the spleen and liver was compared between CTD and vehicle groups. **(H)** HE staining of spleen and liver. The H&E staining of mouse liver, with small focus of extramedullary ematopoiesis (black arrows) in all samples. The H&E staining of mouse spleen, lympho/histiocytic hyperplastic lesion with mitotic figure (black arrows), scattered or clustered, small cell body, low cytoplasm, coarse nuclear chromatin (400×, scale is 50 μm). **(I)** Immunohistochemical staining of CD45, the white arrow points to the positive expression of CD45 which is stained tan (400×, scale is 50 μm). The images were a representative of three independent experiments. Values are presented as the means ± SD. *p < 0.05, **p < 0.01, and ***p < 0.001 *vs* vehicle.

To further investigate the antileukemic effect of CTD *in vivo*, HL-60 cells were injected into the tail vein of NOD/SCID mice after sublethally irradiating with 2.4 Gy. The diagnosis of a leukemia model is difficult, in the early days, we chose to observe the animal’s mental state and whether the body hair was shrunk to initially judge the effect of modeling. Observing the morphological changes of the mice two days after modeling, it can be seen that compared with before modeling, the mice have obvious wrinkles on their body hair and poor mental state (**Supplementary Figures 1A, B**). There is no direct literature reference on CTD acting on the AML model or using NOD/SCID mice. We integrated relevant studies and finally selected the doses of 0.2 and 0.4 mg/kg for the experiments (Huan et al., 2012; Zhang et al., 2017). After 6 weeks of treatment, NOD/SCID mice in the vehicle group were dispirited with low appetite, showed down-bent gait, wrinkled fur, and slow movement, while those in the 0.2 and 0.4 mg/kg of CTD groups behaved more normally, especially in the high dose (0.4 mg/kg) group (**Figure 7A**). The survival curve of the mice was plotted by recording the survival time of each group of mice. As shown in **Figure 7B**, CTD prolonged the survival time of mice in the 0.2 and 0.4 mg/kg of CTD groups compared with those in the vehicle group. Three weeks after the administration, blood indexes related to blood tests were used to judge the validity of the modeling. The determination of blood physiological parameters showed that WBC (**Figure 7C**) decreased, and RBC (**Figure 7D**) increased in both 0.2 and 0.4 (p < 0.01) mg/kg CTD groups compared with those in the vehicle group. Moreover, the hepatomegaly was significantly reduced in mice treated with 0.2 (*p* < 0.05) and 0.4 (*p* < 0.001) mg/kg of CTD compared with those in the vehicle group (**Figures 7E, F**). Meanwhile, the splenomegaly was also remarkably decreased in mice treated with 0.2 (*p* < 0.001) and 0.4 (*p* < 0.001) mg/kg of CTD compared with those in the vehicle group (**Figures 7E, G**). The HE staining results in **Figure 7H** showed that an obvious infiltration was observed in the liver and spleen of mice in the vehicle group, with different degrees of swelling and diffuse growth of tumor cells. Compared with the vehicle group, the histopathological changes were alleviated, and the infiltration was also relieved in the CTD groups. Notably, the 0.2 mg/kg of the CTD group was more obvious than those in the 0.4 mg/kg of CTD group. Immunohistochemical staining results in **Figure 7I** revealed that the expression of CD45, a pan-leukocyte marker, was obviously reduced in 0.2 and 0.4 mg/kg of CTD compared with those in the vehicle group.

## Discussion

Mylabris has been used as a folk medicine for more than 2,000 years with the function of breaking blood and removing phlegm, dispersing and eliminating dysentery, and attacking poisonous acne. It is frequently prescribed in formula for the treatment of various tumors in clinical practice. CTD, the active principle ingredient in Mylabris, has been used as an anticancer agent in China, particularly for hepatoma and oesophageal carcinoma. In this study, our results demonstrated that CTD markedly inhibited the viability of AML cells. HL-60 exhibited the highest sensitivity to CTD with an IC_50_ of 6.21 µM. Moreover, CTD potently induced the apoptosis of HL-60 cells as reflected by flow cytometry analysis and Hoechst staining. Apoptosis of cells can be carried out in a variety of ways. The extrinsic pathway of apoptosis is triggered by the recruitment of corresponding ligands to the intracellular death domain and subsequent activation of initiator caspase 8, which is one of the caspase enzymes critical for the activation of downstream effector caspases (Kumar et al., 2005). Our results showed that CTD promoted the cleavage of caspase 8, followed by the activation of caspase 3 and PARP. Consistently, Efferth T et al. previously demonstrated that CTD caused oxidative stress and subsequently triggered DNA damage and p53-dependent apoptosis in leukemia cells (Efferth et al., 2005; Efferth, 2005).

There is increasing evidence that loss of Nur77 is closely related to the development of AML (Boudreaux et al., 2012). It has been reported that activation of Nur77 pathway is involved in the apoptosis inducing effect of norcantharidin in melanoma cells (Liu et al., 2011). We thereby examined the effect of CTD on Nur77 expression. Our result of Western blotting showed CTD induced the expression of Nur77. Silencing Nur77 reduced the apoptotic rate of CTD-promoted HL-60 cells. This indicates that Nur77 may play a regulatory role in CTD-induced apoptosis of HL-60 cells. One of the pathways of Nur77-mediated apoptosis is related to the mitochondria localization of Nur77 and subsequent Bcl-2 conformational change. It was found that when Nur77 is localized to mitochondria, it can interact with anti-apoptotic Bcl-2, induce a conformational change of Bcl-2 protein by binding ligand binding domain (LBD) to the N-terminal loop of Bcl-2, and promote the exposure of Bcl-2 pro-apoptotic BH3 domain, resulting in the conversion of Bcl-2 from an anti-apoptotic into a proapoptotic molecule (Lin et al., 2004). Therefore, we subsequently investigated the potential effect of CTD on Nur77 translocation and Bcl-2 conformational change. Western blotting showed that CTD obviously increased the enrichment of Nur77 in the mitochondria. It is suggested that CTD-induced apoptosis may be highly correlated with the translocation of Nur77. Furthermore, our immunoprecipitation and flow cytometry results showed that CTD promoted the binding between Nur77 and Bcl-2 and increased the exposure of the BH3 domain of Bcl-2, which indicating Nur77-mediated conformational change and the functional switching of Bcl-2 may be involved in CTD-induced apoptosis.

The multi-step transformation leads to hematopoietic progenitor cells becoming resistant to cell death through unlimited cell cycles. Meanwhile, increased proliferation predisposes these cells to mutations and may contribute to leukemia transformation. In our study, we found that CTD significantly inhibited the proliferation of HL-60 and Kasumi-1 cells and induced the cycle arrest of HL-60 cells at G2/M phase. Each phase of the cell cycle is regulated by several cyclin-dependent kinases (CDKs) that bind to the corresponding cyclins to regulate cell cycle progression (Zhou et al., 2016; Otto and Sicinski, 2017). P21, p27, and p53 act as CDK inhibitors and play a corresponding role in different processes of cell cycle. It has been found that p53 regulates mammalian responses to DNA damage, inhibits G2/M cell cycle, inhibits Cyclin B1 transcription, and plays an important role in controlling tumor development (Innocente et al., 1999). P21 directly controls the cell cycle through p53 and p53-independent pathways and is involved in maintaining G1 cell cycle arrest (El-Deiry, 2016). P27 inhibits the binding of some cyclins to CDKs and prevents the onset of tumors consequently (Roy and Banerjee, 2015). Our Western Blot results showed that CTD up-regulated the expression levels of p53 and p27 in HL-60 cells, but had no significant effect on the expression of p21. In addition, CTD down-regulated the expression levels of cyclin B1, cyclin E, and the cyclin-dependent kinase CDK2. It is indicated that CTD blocked the G2/M phase arrest of HL-60 cells by regulating these cell cycle-related proteins and blocked the abnormal proliferation of HL-60 cells. Accumulating evidence demonstrated that many anticancer compounds trigger apoptosis with G2/M cell cycle arrest. Anticancer drugs that induce G2/M checkpoint regulation disorders can be classified into two categories. An abolition of the G2/M checkpoint affects the entry of damaged cells into mitosis and further induces apoptosis (Kawabe, 2004). Another approach is to promote G2/M checkpoints and subsequently induce apoptosis (Weir et al., 2007). In our study, the results indicate that the increased population of G2/M cells may have a great positive relation with elevated apoptotic population.

Cell cycle arrest is an essential early event in cell differentiation (Chen et al., 2013). Emerging evidence demonstrated that Nur77 played a key role in the differentiation of HSCs and myeloid progenitors (Mullican et al., 2007). Moreover, Nur77 was also found to be a modulator in the differentiation of Ly6C-monocytes (Hanna et al., 2011), suggesting the critical role of Nur77 in differentiation. Thus, we then determined the ability of CTD to induce differentiation in HL-60 cells. Our results of the Wright–Giemsa staining and NBT reduction assay showed that CTD obviously induced the differentiation of HL-60 cells. Such an effect was further confirmed by the detection of myeloid differentiation marker CD11b. Importantly, silencing Nur77 reduced CTD-mediated cell cycle arrest and increase of the rate of NBT positive cells in HL-60 cells and partially attenuated the effect of CTD on the expression of CD11b. Transcription factors c-Jun and Jun B are major members of the AP-1 family (Castellazzi et al., 1991). It has been found that the decrease of c-Jun and Jun B levels in AML mice was correlated with the blockage of differentiation, the increase of self-renewal, and the promotion of the development of AML cells (Steidl et al., 2006). Emerging evidence also indicates that c-Jun and Jun B are the downstream mechanism of Nur77 and related to Nur77-mediated differentiation (Mullican et al., 2007). Our results demonstrated that CTD up-regulated the expression of c-Jun and Jun B. These results indicate that Nur77 is required for the differentiation of CTD-induced acute myeloid leukemia cells.

In conclusion, CTD inhibited the viability of AML cells and induced the apoptosis of HL-60 cells, which was shown to be correlated with the mitochondria localization of Nur77 and subsequent Bcl-2 conformational change. Furthermore, CTD also suppressed the proliferation, induced the G2/M cell cycle arrest and promoted the differentiation of HL-60 cells, which was also found to be related to Nur77-mediated signaling pathway. Together, our study demonstrates that Nur77-mediated signaling pathway played a critical role in the anti-AML effect of CTD.

## Data Availability Statement

The datasets generated for this study are available on request to the corresponding author.

## Ethics Statement

The animal study was reviewed and approved by the Southwestern University Institutional Animal Care and Use Committee.

## Author Contributions

HQ conceived and designed the experiments. ZY, LL, and CW performed the main experiments and analyzed the data. HH, GL, HM, and LP participated in *in vitro* studies. MJ, QL, and PL participated in *in vivo* studies. HQ and ZY wrote the manuscript. All authors contributed to the article and approved the submitted version.

## Funding

This work was supported by Chongqing Health and Planning Commission Traditional Chinese Medicine Science and Technology Project (ZY201702120). Chongqing Research Program of Basic Research and Frontier Technology (no. cstc2018jcyjA1295, cstc2017jcyjAX0299).

## Conflict of Interest

The authors declare that the research was conducted in the absence of any commercial or financial relationships that could be construed as a potential conflict of interest.
