# Supplementary material for: Cantharidin Induces Apoptosis and Promotes Differentiation of AML Cells Through Nuclear Receptor Nur77-Mediated Signaling Pathway
Source: Front Pharmacol. 2020 Aug 28;11:1321. doi: 10.3389/fphar.2020.01321 (PMC7485522; doi:10.3389/fphar.2020.01321)
Supplement: Supplementary file 1 [file Image_1.pdf]

Cantharidin induces apoptosis and promotes differentiation of AML cells through nuclear receptor Nur77-mediated signaling pathway

Zanyang Yu [1], Li Li [1], Chengqiang Wang [1], Hui He [1], Gen Liu [1], Haoyue Ma [1], Lei Pang [1], Mingdong Jiang [2], Qianwei Lu [2], Pan Li [2], Hongyi Qi1 [1*]

[1]College of Pharmaceutical Sciences, Southwest University, 2 Tiansheng Road, Beibei District, Chongqing 400716, China

[2]Radiotherapy Department, Chongqing Ninth People's Hospital, Jialing village 69, 10 Beibei District, Chongqing 400700, China

*** Correspondence:**

Corresponding Author

Prof. H.Q, College of Pharmaceutical Sciences, Southwest University, 2 Tiansheng Road, Beibei District, Chongqing 400716, China

E-mail: hongyiqi@swu.edu.cn.

Tel/Fax: +86 23 68251225.

-- Supplementary Fig.1.

A B

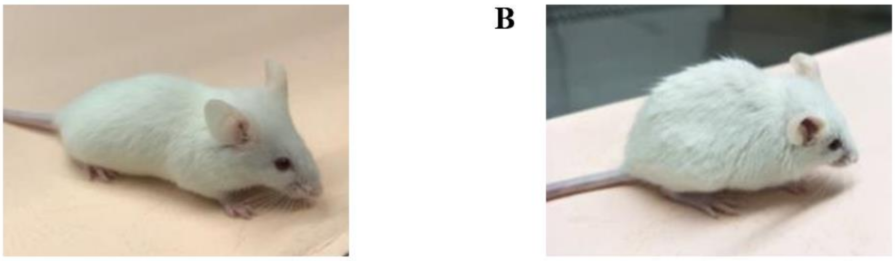

Supplementary Fig.1. (A) The physical status of the normal mice. (B) The physical status of the mice two days after modeling.
